# Determination of the emergency phase for response against endemic disease outbreak: A case of Lassa fever outbreak in Nigeria

**DOI:** 10.7189/jogh.10.020353

**Published:** 2020-12

**Authors:** Oladipupo Ipadeola, Yuki Furuse, Tanyth de Gooyer, Chioma Dan-Nwafor, Geoffrey Namara, Elsie Ilori, Chikwe Ihekweazu

**Affiliations:** 1 Centers for Disease Control and Prevention, Abuja, Nigeria; 2 World Health Organization, Abuja, Nigeria; 3 Kyoto University, Kyoto, Japan; 4 Victorian Department of Health and Human Services, Melbourne, Australia; 5 Nigeria Centre for Disease Control, Abuja, Nigeria

Since 2017, Nigeria has experienced large outbreaks of Lassa fever (LF) [1,2]. The Nigeria Centre for Disease Control (NCDC) activated its Emergency Operations Centre for the outbreak response. However, it was difficult to determine the emergency phase for LF outbreak response because of its endemicity. Because there are ongoing sporadic LF cases throughout the year [2], a single case of the disease cannot be the trigger to determine the emergency phase of LF such as the case of Ebola [3]. The World Health Organization advocated the use of alerts for malaria when weekly cases exceed the 75^th^ percentile of cases from the same week in previous years [4]. However, applying similar thresholds for LF seems too low to implement intensive response in resource-limiting setting. Additionally, the thresholds do not take consideration of the capacity of response activity itself.

Here, we describe the development of composite indicators to determine the emergency phase for LF outbreak response in Nigeria. The composite indicators consist of seven criteria to reflect the outbreak situation from various aspects.

## COMPOSITE INDICATORS FOR EMERGENCY PHASE FOR OUTBREAK RESPONSE

### 1. Number of confirmed cases

Emergency phase designation criteria thresholds were developed using statistical criteria [2]. The thresholds were developed as the mean plus two standard deviations of the weekly number of confirmed cases for the period of 2 weeks before and after a particular week in the past 3 years (eg, data from week 3-7 in 2016-2018 for the development of threshold for week 5 in 2019). This accounts for variation in LF incidence throughout the year and the previous years.

### 2. Number of states with cases exceeding bed capacity

Even if the number of confirmed cases at the national level was below the threshold defined in criterion 1, the outbreak could surpass local health care capacity. Therefore, we monitored the number of cases against bed capacity in LF treatment center at the state level.

### 3. Number of suspected cases

The number of confirmed cases would be less meaningful if there was no adequate surveillance activity to detect LF cases. Therefore, the number of suspected cases including both laboratory-confirmed and laboratory-negative cases was monitored to ascertain sufficient surveillance activity. To consider seasonal fluctuation of the disease, the lower thresholds were developed as the mean minus one standard deviation of the number of suspected cases in the past 3 weeks in the same year.

### 4. Case fatality rate

Timely and appropriate medical care, such as ribavirin administration and renal dialysis, was reported to improve prognosis of patients with LF [1,5]. Unexpectedly high case fatality rate indicates malfunction of the health care system such as delayed referral to treatment center and poor compliance with case management guidelines [5]. We monitored weekly case fatality rate and compared it with that in the previous year.

### 5. Infection among health care workers

Nosocomial human-to-human transmission of the disease can be prevented by appropriate standard precaution strategy. The criterion is met when there are ≥2 confirmed cases among health care workers.

### 6. Safe burial

Unsafe burial is considered to be a mode of secondary transmission of viral hemorrhagic fever. National guidelines stated that a safe and dignified burial should be conducted for every LF corpse by designated safe burial team [5]. There should be no unsafe burial of LF patient.

### 7. Turnaround time of laboratory test

Timely diagnosis of LF is important for better clinical management of patients and for prevention of further spread of the disease. Although in Nigeria, a national network of laboratories covers the entire country for LF diagnosis, the surge of submission of samples during outbreak affected sample transportation and diagnostic test, resulting in the delay in turnaround time for diagnostic results. We monitored the proportion of samples in which the time from sample collection to diagnostic test report was ≥48 hours and compared this proportion with that observed in the previous year.

**Figure:**
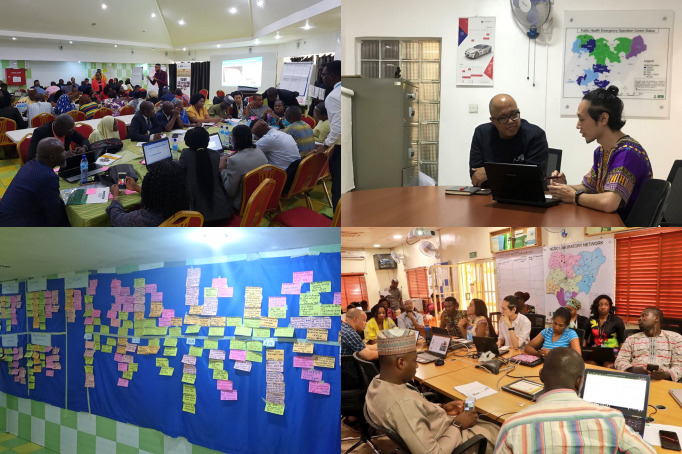
Photo: Meetings on Lassa fever outbreak response with national and local health authorities and partners (from the authors’ own collection, used with permission).

## APPLICATION OF INDICATORS AND INTERPRETATION OF EMERGENCY

Using the seven composite indicators, “Emergency” of the outbreak was defined as either “criterion 1 and one more criterion” or “three or more of criterion 2–7.” **Table 1** shows the situation of LF outbreak in 2019. Emergency phase started from week 3, and the emergency phase was declared over when there was no “emergency” in 4 consecutive weeks (week 22). The NCDC have used this approach to declare the emergency phase and its containment to change intensity of outbreak response. This kind of composite indicators considering various aspects of an outbreak could be useful to determine emergency phase not only for LF but also for other endemic diseases. Determination of emergency phase for outbreak response would enable us to effectively and efficiently allocate resources particularly in resource-limiting settings.

**Table 1:** Composite indicators and situation of Lassa fever outbreak in Nigeria in 2019

			Wk 1	Wk 2		Wk 3			Wk 4			Wk 5			Wk 6			Wk 7			Wk 8			Wk 9			Wk 10			Wk 11			Wk 12			Wk 13			Wk 14			Wk 15			Wk 16			Wk 17			Wk 18			Wk 19			Wk 20			Wk 21			Wk 22			Wk 23			Wk 24		
**Criterion 1**	Indicator	Number of confirmed cases*	25	35		74			77			68			37			25			23			39			52			23			15			16			11			3			6			8			11			4			6			3			3			6			4		
	Threshold	Calculated from data in the past 3 years	38	42		44			48			61			64			64			62			59			44			30			17			16			16			13			10			9			7			7			6			6			5			8			9		
	Criterion met?						**YES**			**YES**			**YES**															**YES**									**YES**															**YES**						**YES**														
**Criterion 2**	Indicator	Number of states with cases exceeding bed capacity	0	0		3			4			3			1			1			1			1			2			1			0			0			0			0			0			0			0			0			0			0			0			0			0		
	Threshold	Fixed	2	2		2			2			2			2			2			2			2			2			2			2			2			2			2			2			2			2			2			2			2			2			2			2		
	Criterion met?						**YES**			**YES**			**YES**															**YES**																																												
**Criterion 3†**	Indicator	Number of suspected cases*	64	130		170			193			217			200			163			119			182			215			152			137			126			88			84			90			59			79			77			100			100			65			67			65		
	Lower threshold	Calculated from data in the past 3 weeks	65	59		48			67			132			169			190			165			120			122			123			151			126			125			91			76			84			61			60			60			72			79			68			57		
	Criterion met?		**YES**																**YES**			**YES**												**YES**			**YES**			**YES**			**YES**						**YES**															**YES**			**YES**					
**Criterion 4**	Indicator	Case fatality rate (%)	28.0	22.9		18.9			14.3			20.6			27.0			24.0			26.1			20.5			21.2			17.4			33.3			12.5			9.1			0			0			12.5			36.4			0			16.7			0			33.3			16.7			25.0		
	Threshold	Data from the previous year	25.0	25.0		25.0			25.0			25.0			25.0			25.0			25.0			25.0			25.0			25.0			25.0			25.0			25.0			25.0			25.0			25.0			25.0			25.0			25.0			25.0			25.0			25.0			25.0		
	Criterion met?		**YES**													**YES**						**YES**												**YES**																		**YES**												**YES**						**YES**		
**Criterion 5**	Indicator	Number of confirmed cases among healthcare workers	0	0		1			1			4			3			1			1			0			0			1			0			1			0			0			0			1			0			0			0			0			0			0			0		
	Threshold	Fixed	2	2		2			2			2			2			2			2			2			2			2			2			2			2			2			2			2			2			2			2			2			2			2			2		
	Criterion met?												**YES**			**YES**																																																								
**Criterion 6**	Indicator	Number of unsafe burial	0	0		0			0			0			0			0			0			0			0			0			0			0			0			0			0			0			0			0			0			0			0			0			0		
	Threshold	Fixed	1	1		1			1			1			1			1			1			1			1			1			1			1			1			1			1			1			1			1			1			1			1			1			1		
	Criterion met?																																																																							
**Criterion 7**	Indicator	Proportion of samples that took ≥48 hours from sample collection to diagnostic test report (%)	20.3	26.0		20.0			17.6			17.9			19.0			19.5			29.5			15.6			10.3			15.2			22.0			17.2			12.0			28.8			14.9			17.5			17.9			12.7			10.9			13.3			15.6			12.3			9.4		
	Threshold	Data from the previous year	22.0	22.0		22.0			22.0			22.0			22.0			22.0			22.0			22.0			22.0			22.0			22.0			22.0			22.0			22.0			22.0			22.0			22.0			22.0			22.0			22.0			22.0			22.0			22.0		
	Criterion met?				**YES**																	**YES**												**YES**									**YES**																													
**Emergency defined by the composite indicators‡**								**YES**			**YES**			**YES**									**YES**						**YES**						**YES**			**YES**															**YES**																			
**Emergency phase‡**								**Start**			**Ong.**			**Ong.**			**Ong.**			**Ong.**			**Ong.**			**Ong.**			**Ong.**			**Ong.**			**Ong.**			**Ong.**			**Ong.**			**Ong.**			**Ong.**			**(End)** §			**Ong.**			**Ong.**			**Ong.**			**Ong.**			**End**							

Wk – week, Ong. – ongoing

*The definition of suspected and confirmed LF cases used for the indicators was described elsewhere [1].

†Criterion 3 is met when indicator is ≤ the thresholds. For others, each criterion is met when indicator is ≥ the thresholds.

‡Interpretation of composite indicators to define emergency phase is explained in detail in the main texts.

§According to the definition, week 17 should have been the end of the emergency phase. However, the containment of emergency phase was not declared then because resurge of the number of cases was recognized during week 18 before completion of assessment for week 17.
